# *N*-(3,4-Dichloro­phen­yl)-2,4-dimethyl­benzene­sulfonamide

**DOI:** 10.1107/S1600536809028840

**Published:** 2009-07-25

**Authors:** B. Thimme Gowda, Sabine Foro, P. G. Nirmala, Hartmut Fuess

**Affiliations:** a Department of Chemistry, Mangalore University, Mangalagangotri 574 199, Mangalore, India; b Institute of Materials Science, Darmstadt University of Technology, Petersenstrasse 23, D-64287 Darmstadt, Germany

## Abstract

In the crystal structure of the title compound, C_14_H_13_Cl_2_NO_2_S, the configurations of the N—C bond with respect to the S=O bonds are *trans* and *gauche*. The mol­ecule is bent at the S atom with a C—SO_2_—NH—C torsion angle of −69.7 (2)°. The conformation of the N—H bond is *syn* to the 3-chloro group in the substituted aniline ring. The two benzene rings are tilted with respect to each other by 82.4 (1)°. The presence of N—H⋯O(S) hydrogen bonding packs the mol­ecules into supra­molecular chains along the *b* axis.

## Related literature

For our study of the effect of substituents on the structures of *N*-(ar­yl)-aryl­sulfonamides, see: Gowda *et al.* (2008 ▶; 2009**a* ▶,b* ▶). For related structures, see: Gelbrich *et al.* (2007 ▶); Perlovich *et al.* (2006 ▶).
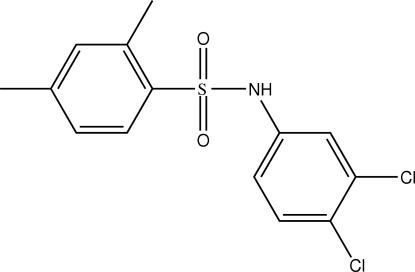


## Experimental

### 

#### Crystal data

C_14_H_13_Cl_2_NO_2_S
*M* *_r_* = 330.21
Monoclinic, 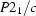

*a* = 8.8046 (7) Å
*b* = 9.2688 (8) Å
*c* = 18.947 (1) Å
β = 99.644 (8)°
*V* = 1524.4 (2) Å^3^
*Z* = 4
Mo *K*α radiation
μ = 0.56 mm^−1^
*T* = 299 K
0.44 × 0.40 × 0.38 mm

#### Data collection

Oxford Diffraction Xcalibur diffractometer with a Sapphire CCD detector
Absorption correction: multi-scan (*CrysAlis RED*; Oxford Diffraction, 2009 ▶) *T*_min_ = 0.790, *T*_max_ = 0.815
10274 measured reflections
3064 independent reflections
2618 reflections with *I* > 2σ(*I*)
*R*_int_ = 0.013

#### Refinement

*R*[*F* ^2^ > 2σ(*F* ^2^)] = 0.037
*wR*(*F* ^2^) = 0.100
*S* = 1.05
3064 reflections
186 parameters
1 restraint
H atoms treated by a mixture of independent and constrained refinement
Δρ_max_ = 0.27 e Å^−3^
Δρ_min_ = −0.39 e Å^−3^

### 

Data collection: *CrysAlis CCD* (Oxford Diffraction, 2009 ▶); cell refinement: *CrysAlis RED* (Oxford Diffraction, 2009 ▶); data reduction: *CrysAlis RED*; program(s) used to solve structure: *SHELXS97* (Sheldrick, 2008 ▶); program(s) used to refine structure: *SHELXL97* (Sheldrick, 2008 ▶); molecular graphics: *PLATON* (Spek, 2009 ▶); software used to prepare material for publication: *SHELXL97*.

## Supplementary Material

Crystal structure: contains datablocks I, global. DOI: 10.1107/S1600536809028840/tk2510sup1.cif

Structure factors: contains datablocks I. DOI: 10.1107/S1600536809028840/tk2510Isup2.hkl

Additional supplementary materials:  crystallographic information; 3D view; checkCIF report

## Figures and Tables

**Table 1:** Hydrogen-bond geometry (Å, °)

*D*—H⋯*A*	*D*—H	H⋯*A*	*D*⋯*A*	*D*—H⋯*A*
N1—H1*N*⋯O1^i^	0.833 (16)	2.176 (17)	2.984 (2)	164 (2)

Symmetry code: (i) 
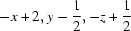
.

**Fig. 1.**
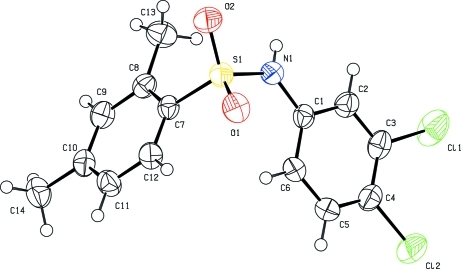
Molecular structure of (I), showing the atom labelling scheme and displacement ellipsoids are drawn at the 50% probability level

## supplementary crystallographic information

### Comment

As part of a study of substituent effects on the structures of *N*-(aryl)-arylsulfonamides (Gowda *et al.*, 2008, 2009*a*, *b*), in the present work, the structure of 2,4-dimethyl-*N*-(3,4-dichlorophenyl)benzenesulfonamide (I) has been determined. The conformations of the N—C bond in the C—SO_2_—NH—C segment are *trans* and *gauche* to the S=O bonds (Fig. 1). The molecule is bent at the S atom with the C—SO_2_—NH—C torsion angle of -69.7 (2)°, compared to the values of -48.2 (2)° in 2,4-dichloro-*N*-(3,4-dichlorophenyl)benzenesulfonamide (II) (Gowda *et al.*, 2009*b*), and 46.1 (3)°  and 47.7 (3)° in the two independent molecules of 2,4-dimethyl-*N*-(phenyl)benzenesulfonamide (III) (Gowda *et al.*, 2009*a*). The conformation of the N—H bond is *syn* to the *meta*-chloro group in the substituted aniline ring. The two benzene rings in (I) are tilted by 82.4 (1)° to each other compared to the values of 68.9 (1)° in II, and 67.5 (1)° and 72.9 (1)° in III. The other bond parameters in (I) are similar to those observed in II, III, and other aryl sulfonamides (Gowda *et al.*, 2008; Perlovich *et al.*, 2006; Gelbrich *et al.*, 2007). The crystal packing of molecules in (I) is *via* N—H···O(S) hydrogen bonding (Table 1) leading to a supramolecular chain.

### Experimental

A solution of 1,3-xylene (10 ml) in chloroform (40 ml) was treated dropwise with chlorosulfonic acid (25 ml) at 0 ° C. After the initial evolution of hydrogen chloride subsided, the reaction mixture was brought to room temperature and poured into crushed ice in a beaker. The chloroform layer was separated, washed with cold water and allowed to evaporate slowly. The residual 2,4-dimethylbenzenesulfonylchloride was treated with 3,4-dichloroaniline in a stoichiometric ratio and boiled for ten minutes. The reaction mixture was then cooled to room temperature and added to ice-cold water (100 ml). The resultant solid, 2,4-dimethyl-*N*-(3,4-dichlorophenyl)benzenesulfonamide, was filtered under suction and washed thoroughly with cold water. It was then recrystallized to constant melting point from dilute ethanol. The prisms used in the X-ray analysis were grown in ethanolic solution by a slow evaporation at room temperature.

### Refinement

The H atom of the NH group was located in difference map and was refined with restrained geometry to 0.86 (2) Å. The other H atoms were positioned with idealized geometry using a riding model [C—H = 0.93—0.96 Å], and were refined with isotropic displacement parameters set to 1.2 times of the *U*_eq_ of the parent atom.

### Crystal data

**Table:**

C_14_H_13_Cl_2_NO_2_S	*F*(000) = 680
*M**_r_* = 330.21	*D*_x_ = 1.439 Mg m^−^^3^
Monoclinic, *P*2_1_/*c*	Mo *K*α radiation, λ = 0.71073 Å
Hall symbol: -P 2ybc	Cell parameters from 4751 reflections
*a* = 8.8046 (7) Å	θ = 3.0–27.6°
*b* = 9.2688 (8) Å	µ = 0.56 mm^−^^1^
*c* = 18.947 (1) Å	*T* = 299 K
β = 99.644 (8)°	Prism, colourless
*V* = 1524.4 (2) Å^3^	0.44 × 0.40 × 0.38 mm
*Z* = 4	

### Data collection

**Table:**

Oxford Diffraction Xcalibur diffractometer with a Sapphire CCD detector	3064 independent reflections
Radiation source: fine-focus sealed tube	2618 reflections with *I* > 2σ(*I*)
graphite	*R*_int_ = 0.013
Rotation method data acquisition using ω and φ scans	θ_max_ = 26.4°, θ_min_ = 2.9°
Absorption correction: multi-scan (*CrysAlis RED*; Oxford Diffraction, 2009)	*h* = −9→10
*T*_min_ = 0.790, *T*_max_ = 0.815	*k* = −11→11
10274 measured reflections	*l* = −23→23

### Refinement

**Table:**

Refinement on *F*^2^	Primary atom site location: structure-invariant direct methods
Least-squares matrix: full	Secondary atom site location: difference Fourier map
*R*[*F*^2^ > 2σ(*F*^2^)] = 0.037	Hydrogen site location: inferred from neighbouring sites
*wR*(*F*^2^) = 0.100	H atoms treated by a mixture of independent and constrained refinement
*S* = 1.05	*w* = 1/[σ^2^(*F*_o_^2^) + (0.0506*P*)^2^ + 0.7353*P*] where *P* = (*F*_o_^2^ + 2*F*_c_^2^)/3
3064 reflections	(Δ/σ)_max_ = 0.007
186 parameters	Δρ_max_ = 0.27 e Å^−^^3^
1 restraint	Δρ_min_ = −0.39 e Å^−^^3^

### Special details

**Table:**

Experimental. CrysAlis RED (Oxford Diffraction, 2009) Empirical absorption correction using spherical harmonics, implemented in SCALE3 ABSPACK scaling algorithm.
Geometry. All e.s.d.'s (except the e.s.d. in the dihedral angle between two l.s. planes) are estimated using the full covariance matrix. The cell e.s.d.'s are taken into account individually in the estimation of e.s.d.'s in distances, angles and torsion angles; correlations between e.s.d.'s in cell parameters are only used when they are defined by crystal symmetry. An approximate (isotropic) treatment of cell e.s.d.'s is used for estimating e.s.d.'s involving l.s. planes.
Refinement. Refinement of *F*^2^ against ALL reflections. The weighted *R*-factor *wR* and goodness of fit *S* are based on *F*^2^, conventional *R*-factors *R* are based on *F*, with *F* set to zero for negative *F*^2^. The threshold expression of *F*^2^ > σ(*F*^2^) is used only for calculating *R*-factors(gt) *etc*. and is not relevant to the choice of reflections for refinement. *R*-factors based on *F*^2^ are statistically about twice as large as those based on *F*, and *R*- factors based on ALL data will be even larger.

### Fractional atomic coordinates and isotropic or equivalent isotropic displacement parameters (Å^2^)

**Table:**

	*x*	*y*	*z*	*U*_iso_*/*U*_eq_	
C1	0.7751 (2)	0.1105 (2)	0.26937 (9)	0.0342 (4)	
C2	0.7595 (2)	0.0272 (2)	0.32852 (10)	0.0405 (4)	
H2	0.8407	−0.0308	0.3497	0.049*	
C3	0.6243 (2)	0.0301 (2)	0.35611 (10)	0.0425 (5)	
C4	0.5020 (2)	0.1148 (2)	0.32506 (10)	0.0425 (5)	
C5	0.5178 (2)	0.1972 (3)	0.26616 (10)	0.0472 (5)	
H5	0.4360	0.2544	0.2449	0.057*	
C6	0.6528 (2)	0.1963 (2)	0.23813 (10)	0.0441 (5)	
H6	0.6618	0.2529	0.1985	0.053*	
C7	0.8600 (2)	0.1276 (2)	0.09846 (9)	0.0334 (4)	
C8	0.8733 (2)	−0.0147 (2)	0.07500 (10)	0.0363 (4)	
C9	0.7833 (2)	−0.0518 (2)	0.01025 (10)	0.0410 (4)	
H9	0.7907	−0.1453	−0.0067	0.049*	
C10	0.6828 (2)	0.0432 (2)	−0.03070 (10)	0.0403 (4)	
C11	0.6726 (2)	0.1824 (2)	−0.00542 (10)	0.0434 (5)	
H11	0.6057	0.2479	−0.0317	0.052*	
C12	0.7608 (2)	0.2250 (2)	0.05836 (10)	0.0406 (4)	
H12	0.7537	0.3191	0.0746	0.049*	
C13	0.9776 (3)	−0.1262 (2)	0.11551 (13)	0.0515 (5)	
H13A	0.9396	−0.1519	0.1584	0.062*	
H13B	1.0797	−0.0874	0.1277	0.062*	
H13C	0.9800	−0.2103	0.0862	0.062*	
C14	0.5881 (3)	−0.0036 (3)	−0.10031 (11)	0.0529 (5)	
H14A	0.5855	−0.1071	−0.1026	0.064*	
H14B	0.6331	0.0336	−0.1394	0.064*	
H14C	0.4851	0.0328	−0.1035	0.064*	
N1	0.91696 (19)	0.1015 (2)	0.24438 (8)	0.0407 (4)	
H1N	0.976 (2)	0.035 (2)	0.2605 (12)	0.049*	
O1	0.92732 (17)	0.33947 (15)	0.18616 (7)	0.0471 (4)	
O2	1.12802 (16)	0.15602 (19)	0.18124 (8)	0.0530 (4)	
Cl1	0.61158 (9)	−0.07351 (7)	0.43079 (4)	0.0753 (2)	
Cl2	0.33330 (7)	0.12189 (9)	0.36022 (3)	0.0680 (2)	
S1	0.96962 (5)	0.19135 (5)	0.17881 (2)	0.03717 (14)	

### Atomic displacement parameters (Å^2^)

**Table:**

	*U*^11^	*U*^22^	*U*^33^	*U*^12^	*U*^13^	*U*^23^
C1	0.0378 (10)	0.0348 (9)	0.0301 (8)	−0.0019 (7)	0.0057 (7)	−0.0059 (7)
C2	0.0482 (11)	0.0329 (10)	0.0418 (10)	0.0070 (8)	0.0121 (8)	0.0008 (8)
C3	0.0561 (12)	0.0323 (10)	0.0428 (10)	−0.0013 (9)	0.0190 (9)	−0.0015 (8)
C4	0.0378 (10)	0.0490 (12)	0.0424 (10)	−0.0053 (9)	0.0116 (8)	−0.0117 (9)
C5	0.0372 (11)	0.0635 (14)	0.0389 (10)	0.0079 (9)	0.0010 (8)	−0.0018 (9)
C6	0.0414 (11)	0.0577 (13)	0.0325 (9)	0.0056 (9)	0.0045 (8)	0.0039 (9)
C7	0.0347 (9)	0.0356 (10)	0.0313 (8)	−0.0044 (7)	0.0093 (7)	0.0007 (7)
C8	0.0373 (9)	0.0337 (9)	0.0398 (9)	−0.0012 (8)	0.0121 (7)	0.0019 (7)
C9	0.0476 (11)	0.0346 (10)	0.0421 (10)	−0.0028 (8)	0.0112 (8)	−0.0050 (8)
C10	0.0438 (11)	0.0453 (11)	0.0330 (9)	−0.0057 (9)	0.0100 (8)	−0.0020 (8)
C11	0.0520 (12)	0.0418 (11)	0.0353 (9)	0.0052 (9)	0.0037 (8)	0.0059 (8)
C12	0.0517 (11)	0.0336 (10)	0.0370 (9)	0.0028 (8)	0.0087 (8)	0.0008 (8)
C13	0.0532 (13)	0.0401 (11)	0.0588 (13)	0.0051 (10)	0.0027 (10)	0.0035 (10)
C14	0.0577 (13)	0.0602 (14)	0.0391 (11)	−0.0061 (11)	0.0032 (9)	−0.0050 (10)
N1	0.0381 (9)	0.0497 (10)	0.0350 (8)	0.0079 (7)	0.0080 (7)	0.0061 (7)
O1	0.0542 (9)	0.0374 (8)	0.0481 (8)	−0.0112 (6)	0.0038 (6)	−0.0065 (6)
O2	0.0326 (7)	0.0724 (11)	0.0544 (9)	−0.0062 (7)	0.0087 (6)	−0.0024 (7)
Cl1	0.0973 (5)	0.0599 (4)	0.0818 (5)	0.0169 (3)	0.0531 (4)	0.0292 (3)
Cl2	0.0443 (3)	0.0965 (5)	0.0679 (4)	−0.0024 (3)	0.0234 (3)	−0.0037 (3)
S1	0.0339 (2)	0.0413 (3)	0.0365 (2)	−0.00631 (19)	0.00630 (18)	−0.00184 (19)

### Geometric parameters (Å, °)

**Table:**

C1—C2	1.387 (3)	C9—C10	1.389 (3)
C1—C6	1.389 (3)	C9—H9	0.9300
C1—N1	1.410 (2)	C10—C11	1.385 (3)
C2—C3	1.379 (3)	C10—C14	1.501 (3)
C2—H2	0.9300	C11—C12	1.380 (3)
C3—C4	1.382 (3)	C11—H11	0.9300
C3—Cl1	1.729 (2)	C12—H12	0.9300
C4—C5	1.378 (3)	C13—H13A	0.9600
C4—Cl2	1.7283 (19)	C13—H13B	0.9600
C5—C6	1.381 (3)	C13—H13C	0.9600
C5—H5	0.9300	C14—H14A	0.9600
C6—H6	0.9300	C14—H14B	0.9600
C7—C12	1.391 (3)	C14—H14C	0.9600
C7—C8	1.403 (3)	N1—S1	1.6264 (17)
C7—S1	1.7623 (18)	N1—H1N	0.833 (16)
C8—C9	1.387 (3)	O1—S1	1.4353 (15)
C8—C13	1.505 (3)	O2—S1	1.4258 (15)
			
C2—C1—C6	119.25 (18)	C9—C10—C14	121.03 (19)
C2—C1—N1	116.83 (17)	C12—C11—C10	120.66 (18)
C6—C1—N1	123.92 (17)	C12—C11—H11	119.7
C3—C2—C1	120.29 (18)	C10—C11—H11	119.7
C3—C2—H2	119.9	C11—C12—C7	120.17 (18)
C1—C2—H2	119.9	C11—C12—H12	119.9
C2—C3—C4	120.68 (18)	C7—C12—H12	119.9
C2—C3—Cl1	118.55 (16)	C8—C13—H13A	109.5
C4—C3—Cl1	120.77 (15)	C8—C13—H13B	109.5
C5—C4—C3	118.87 (18)	H13A—C13—H13B	109.5
C5—C4—Cl2	120.05 (16)	C8—C13—H13C	109.5
C3—C4—Cl2	121.06 (16)	H13A—C13—H13C	109.5
C4—C5—C6	121.20 (19)	H13B—C13—H13C	109.5
C4—C5—H5	119.4	C10—C14—H14A	109.5
C6—C5—H5	119.4	C10—C14—H14B	109.5
C5—C6—C1	119.71 (18)	H14A—C14—H14B	109.5
C5—C6—H6	120.1	C10—C14—H14C	109.5
C1—C6—H6	120.1	H14A—C14—H14C	109.5
C12—C7—C8	121.01 (17)	H14B—C14—H14C	109.5
C12—C7—S1	117.22 (14)	C1—N1—S1	127.44 (14)
C8—C7—S1	121.76 (14)	C1—N1—H1N	117.1 (16)
C9—C8—C7	116.59 (17)	S1—N1—H1N	114.8 (16)
C9—C8—C13	119.36 (18)	O2—S1—O1	119.00 (9)
C7—C8—C13	124.05 (18)	O2—S1—N1	105.08 (9)
C8—C9—C10	123.55 (18)	O1—S1—N1	107.69 (9)
C8—C9—H9	118.2	O2—S1—C7	109.99 (9)
C10—C9—H9	118.2	O1—S1—C7	106.92 (9)
C11—C10—C9	118.01 (18)	N1—S1—C7	107.66 (9)
C11—C10—C14	120.96 (19)		
			
C6—C1—C2—C3	−0.2 (3)	C8—C9—C10—C11	0.2 (3)
N1—C1—C2—C3	−179.96 (17)	C8—C9—C10—C14	−179.83 (18)
C1—C2—C3—C4	0.5 (3)	C9—C10—C11—C12	0.4 (3)
C1—C2—C3—Cl1	−179.00 (14)	C14—C10—C11—C12	−179.54 (19)
C2—C3—C4—C5	−0.3 (3)	C10—C11—C12—C7	−0.7 (3)
Cl1—C3—C4—C5	179.16 (16)	C8—C7—C12—C11	0.3 (3)
C2—C3—C4—Cl2	−178.74 (15)	S1—C7—C12—C11	179.45 (15)
Cl1—C3—C4—Cl2	0.7 (2)	C2—C1—N1—S1	−177.17 (15)
C3—C4—C5—C6	−0.1 (3)	C6—C1—N1—S1	3.1 (3)
Cl2—C4—C5—C6	178.33 (16)	C1—N1—S1—O2	173.04 (16)
C4—C5—C6—C1	0.4 (3)	C1—N1—S1—O1	45.23 (19)
C2—C1—C6—C5	−0.2 (3)	C1—N1—S1—C7	−69.74 (18)
N1—C1—C6—C5	179.52 (18)	C12—C7—S1—O2	−129.57 (15)
C12—C7—C8—C9	0.3 (3)	C8—C7—S1—O2	49.56 (17)
S1—C7—C8—C9	−178.82 (13)	C12—C7—S1—O1	0.97 (17)
C12—C7—C8—C13	−179.77 (19)	C8—C7—S1—O1	−179.90 (14)
S1—C7—C8—C13	1.1 (3)	C12—C7—S1—N1	116.45 (15)
C7—C8—C9—C10	−0.6 (3)	C8—C7—S1—N1	−64.42 (16)
C13—C8—C9—C10	179.49 (19)		

### Hydrogen-bond geometry (Å, °)

**Table:**

*D*—H···*A*	*D*—H	H···*A*	*D*···*A*	*D*—H···*A*
N1—H1N···O1^i^	0.83 (2)	2.18 (2)	2.984 (2)	164 (2)

Symmetry codes: (i) −*x*+2, *y*−1/2, −*z*+1/2.
